# Supplementary material for: Structural and biochemical basis for DNA and RNA catalysis by human Topoisomerase 3β
Source: Nat Commun. 2022 Aug 9;13:4656. doi: 10.1038/s41467-022-32221-3 (PMC9363430; doi:10.1038/s41467-022-32221-3)
Supplement: Supplementary file 1 — Supplementary Information [file 41467_2022_32221_MOESM1_ESM.pdf]

Supplementary Information for

Structural and biochemical basis for DNA and RNA catalysis by human Topoisomerase 3β

Xi Yang[1], Sourav Saha[1], Wei Yang[2], Keir Neuman[3] and Yves Pommier[1] ✉

[1] Developmental Therapeutics Branch & Laboratory of Molecular Pharmacology, Center for Cancer Research, National Cancer Institute, NIH, Bethesda, MD 20892, USA.
[2] Laboratory of Molecular Biology, National Institute of Diabetes and Digestive and Kidney Diseases, NIH, Bethesda, MD 20892, USA.
[3] Laboratory of Single Molecule Biophysics, National Heart, Lung and Blood Institute, National Institutes of Health, Bethesda, MD, USA.
✉ email: pommier@nih.gov

Contents:

Supplementary Figures 1-12 and legends.

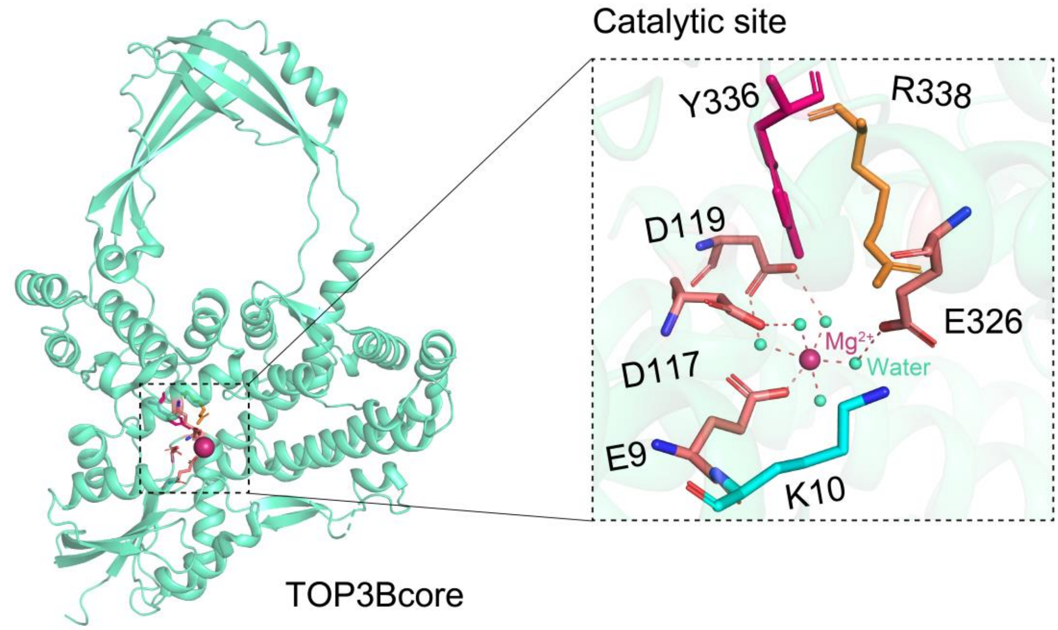

Supplementary figure 1. Divalent metal bound to the TOP3B catalytic center. Crystal structure of the TOP3Bcore apo protein (PDB ID: 5gvc) with a magnesium ion (red-violet sphere) coordinated by four acidic residues. Key residues for TOP3B catalysis are shown in sticks. Blue cyan spheres indicate metal coordinated water molecules.

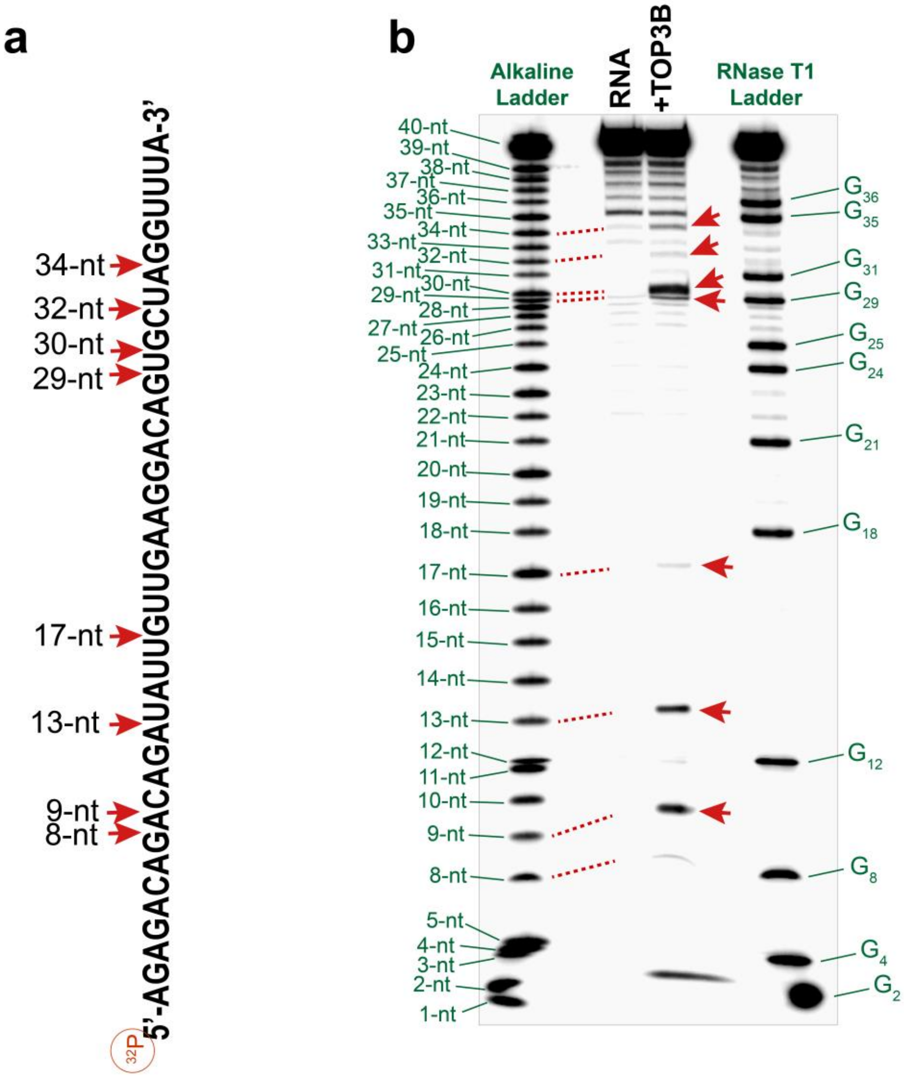

Supplementary figure 2. Mapping of RNA cleavage sites with alkaline hydrolysis ladder and RNase T1 ladder. a, Sequence of the ^{32}P-labeled 40-nt RNA substrate used in the TOP3B cleavage assay. Red arrows indicate the determined cleavage sites of TOP3B. **b**, Analysis of TOP3B cleavages sites in the 40-nt RNA substrate using an alkaline hydrolysis RNA ladder (generated in 50 mM sodium carbonate pH 9.2, 1 mM EDTA), and an RNase T1 ladder (obtained with 1 U RNase T1 in 25 mM sodium citrate pH 5.3, 7M Urea, 1 mM EDTA) on a denaturing polyacrylamide gel. RNA fragments generated via random hydrolysis (alkaline ladder) and G-specific digestions (RNase T1 ladder) are indicated by numbers. Note that RNA ladder bands migrate slightly faster than the TOP3B cleavage products, due to an additional phosphate at their 3'-ends. Assay was repeated twice independently with similar results.

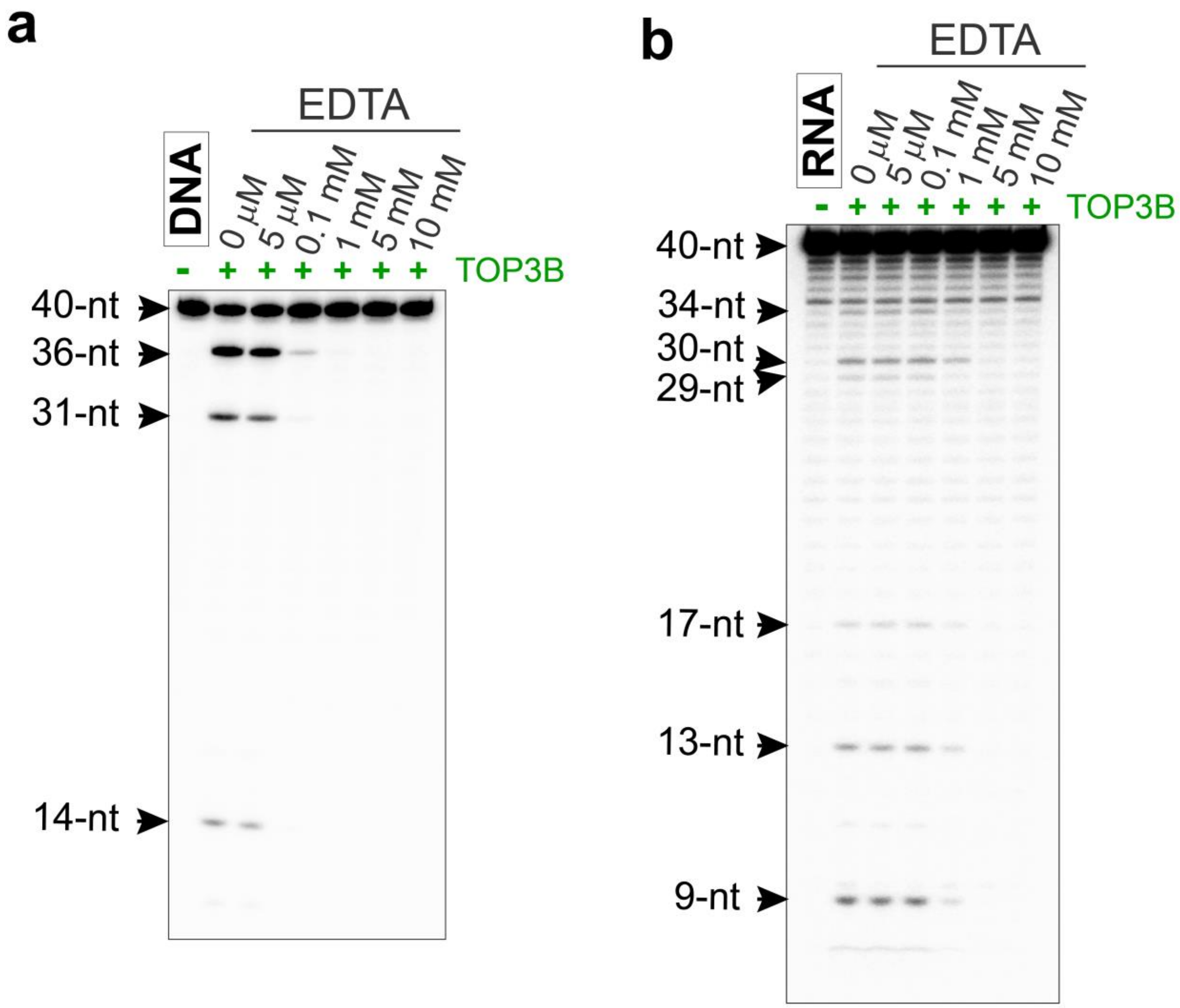

Supplementary figure 3. Excess EDTA suppresses both DNA and RNA cleavage by TOP3B. a, DNA cleavage by TOP3B in the absence and presence of the indicated concentrations of EDTA. Arrows indicate DNA substrate and cleavage products. **b**, RNA cleavage by TOP3B at the indicated EDTA concentrations. Arrows indicate RNA substrate and major cleavage products. Cleavage assays in **a** and **b** were repeated twice independently with similar results.

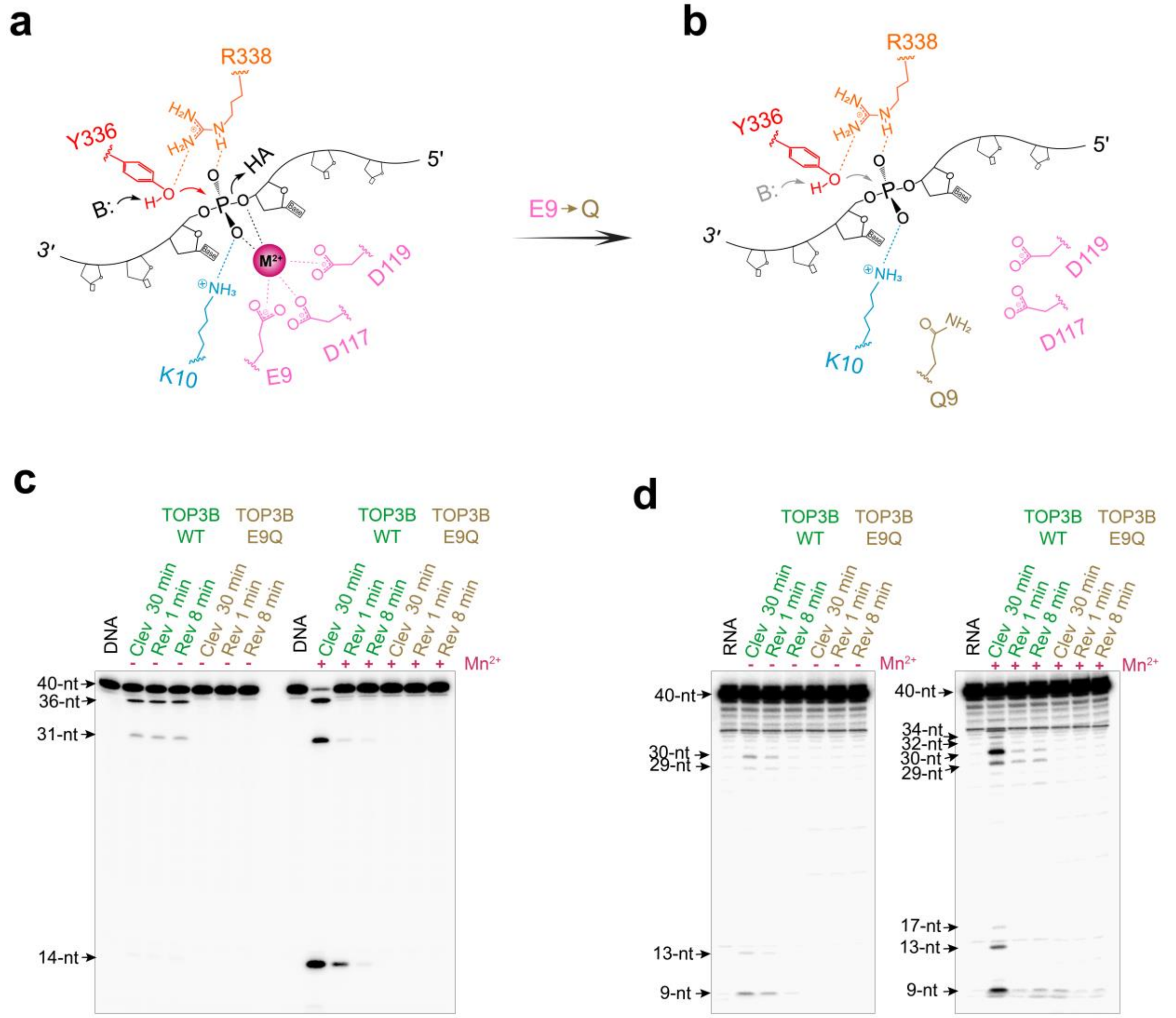

Supplementary figure 4. E9 substitution suppresses DNA and RNA cleavage activities of TOP3B. a, Schematic illustration showing that E9 is part of the divalent cation binding motif of TOP3B, crucial for initiating DNA cleavage. **b**, Replacing E9 to glutamine may lead to loss of metal ion binding and DNA/RNA cleavage. **c**, E9 replacement annihilates DNA cleavage by TOP3B. DNA cleavage and high-salt reversal assays with TOP3B and TOP3B-E9Q were carried out in the absence of divalent metal (with 5 μM EDTA) or presence of 0.1 mM Mn^{2+}. **d**, RNA cleavage activity of TOP3B is suppressed by E9 replacement. RNA cleavage and high-salt reversal assays were conducted using the same divalent-metal/EDTA concentrations as in **c**. Cleavage assays in **c** and **d** were repeated twice independently with similar results.

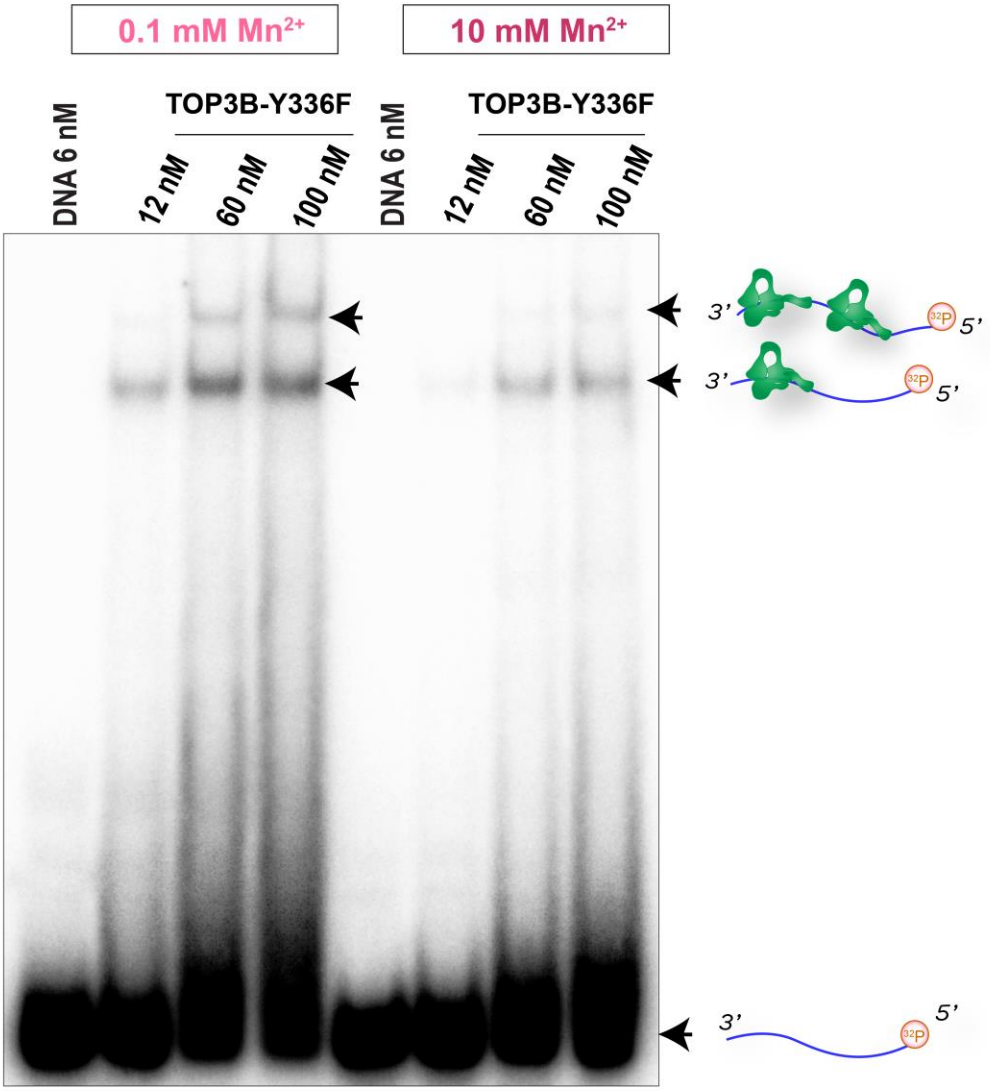

Supplementary figure 5. Excess amount of divalent metal inhibits TOP3B (catalytic inactive mutant TOP3B-Y336F) binding to DNA. EMSA assays were carried out in the presence of 0.1 mM and 10 mM Mn^{2+} using a ^{32}P-labeled 30-nt DNA oligonucleotide. Retardation bands representing enzyme-substrate complexes and free DNA bands are indicated by arrows and cartoons. Assays was repeated twice independently with similar results. Assay was repeated twice independently with similar results.

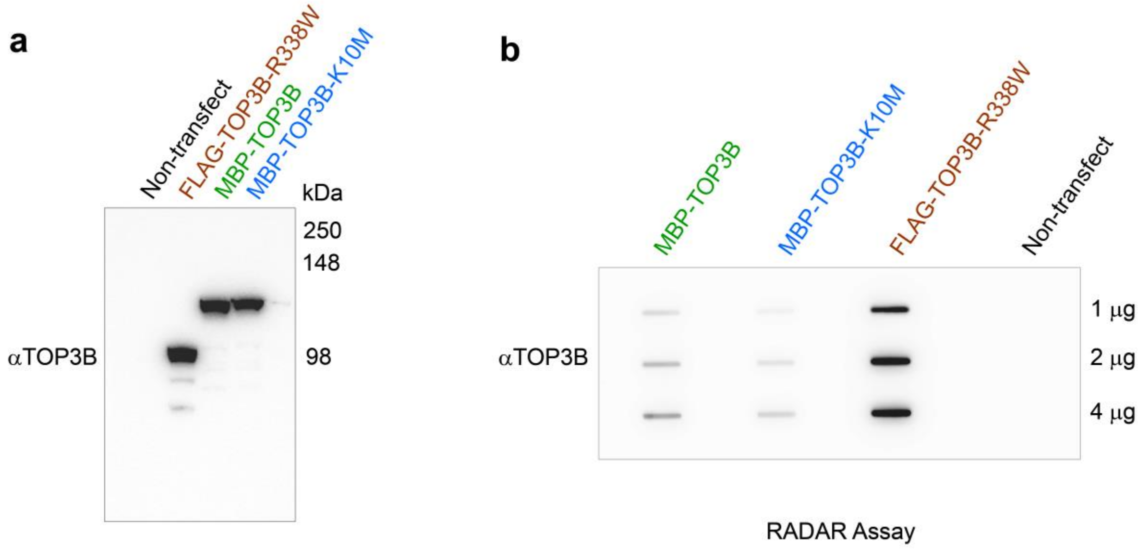

Supplementary figure 6. The TOP3B-K10M mutant shows deficient cleavage complex formation in cells compared to WT TOP3B and the self-trapping mutant TOP3B-R338W[1]. a, Western-blot detection of ectopic expression of recombinant FLAG-TOP3B-R338W, MBP-TOP3B and MBP-TOP3B-K10M after transfections of plasmids containing the corresponding recombinant genes for 72 h. **b,** Detection of TOP3B cleavage complexes in cells by RADAR (Rapid Approach to DNA Adduct Recovery) assay[1] after transfection with plasmids containing recombinant genes of MBP-TOP3B, FLAG-TOP3B-R338W and MBP-TOP3B-K10M for 72 h. Slot blotted TOP3B cleavage complexes (containing both DNA and RNA covalent complexes) were detected with anti-TOP3B antibody. Numbers indicate the amounts of nucleic acids loaded in each slot. Assay was repeated twice independently with similar results.

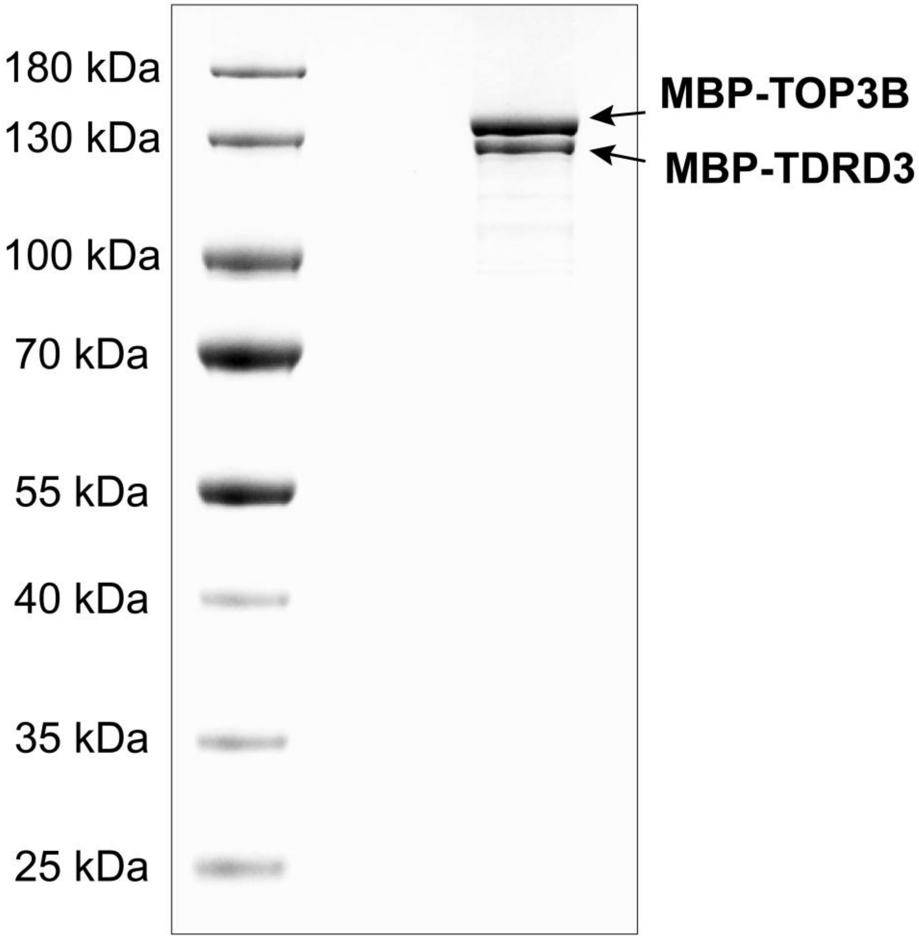

Supplementary figure 7. SDS-PAGE showing formation of a stable heterodimer of MBP-tagged TOP3B and TDRD3 (~1:1 molar ratio) collected from an individual peak during size exclusion gel filtration chromatography. MBP-tagged TOP3B and TDRD3 were pre-incubated in solution for 30 min, concentrated via ultrafiltration, and run down a gel filtration column. Arrows indicate TOP3B and TDRD3 bands collected from an individual elution peak. Numbers indicate the molecular mass (kDa) of the protein standards. Assay was repeated twice independently with similar results.

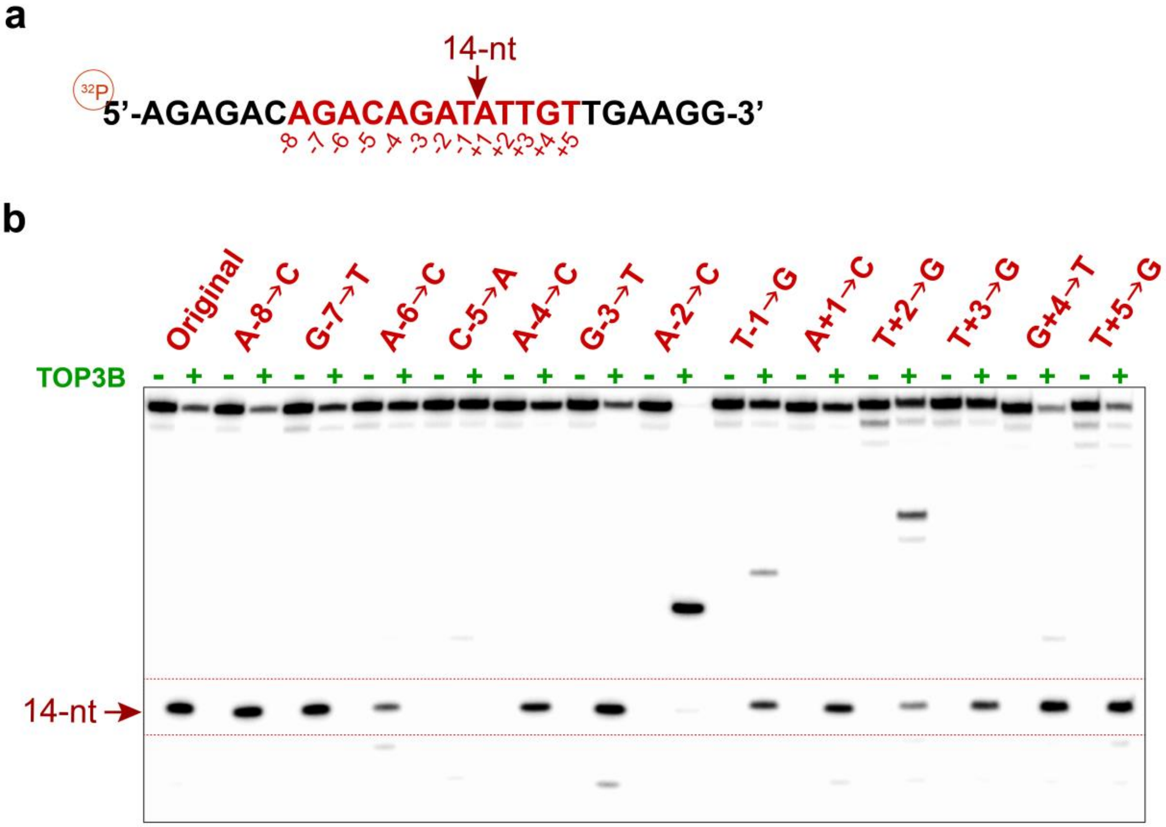

Supplementary figure 8. Single-base substitutions within a TOP3B recognition site alter the DNA cleavage profile of TOP3B. a, Sequence of a 25-nt DNA substrate containing a TOP3B strong cleavage site (red arrow). The number under each DNA base represents their base positions relative to the cleavage site. **b**, Single-base replacements from -6 to +3 positions result in: 1) reduced efficiency of TOP3B cleavage at the original cleavage site (14-nt site), and/or 2) generation of additional enzyme cleavage sites. The 14-nt cleavage products are indicated by a red dotted box and DNA bands outside the box represent products of TOP3B cleavage at other sites. Assay was repeated twice independently with similar results.

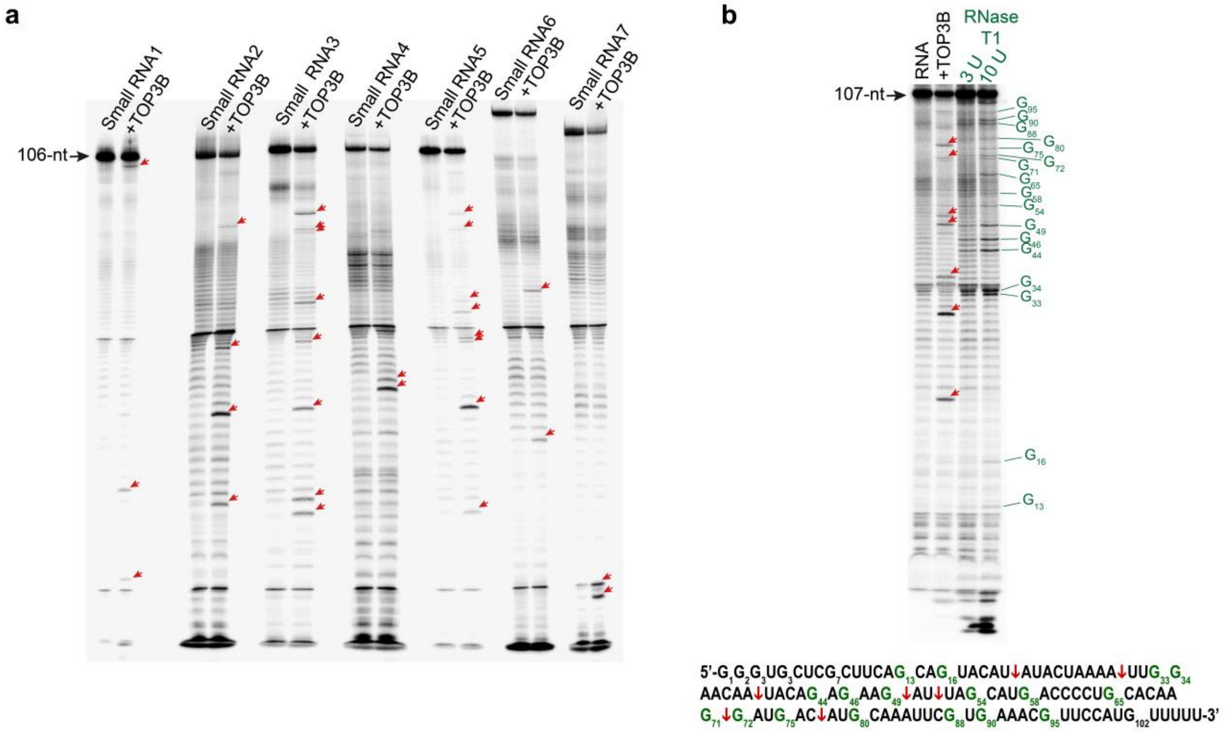

Supplementary figure 9. Mapping TOP3B-mediated RNA cleavage sites with human small RNAs. a, Seven human small RNAs (potential *in vivo* TOP3B targets) were selected and *in vitro* transcribed to generate substrates for the TOP3B cleavage assays. TOP3B cleavage products generated with these RNA substrates are indicated by red arrows. Gene names for Small RNAs 1-7 are listed as follows (in order): MIR1248, RNU6-314P, RNU6-766P, RNU6-988P, RNU6-476P, SNORA51, and MIR6132. **b**, Representative gel showing the mapping of RNA cleavage sites of TOP3B in one of the RNA substrates (Small RNA5). Red arrows indicate major TOP3B cleavage sites. Numbers indicate guanine (G)-specific digestion products by RNase T1. The sequence of the RNA substrate is provided below the gel and the TOP3B cleavage sites determined from the gel are indicated by red arrows. Cleavage assays in **a** and **b** were repeated twice independently with similar results.

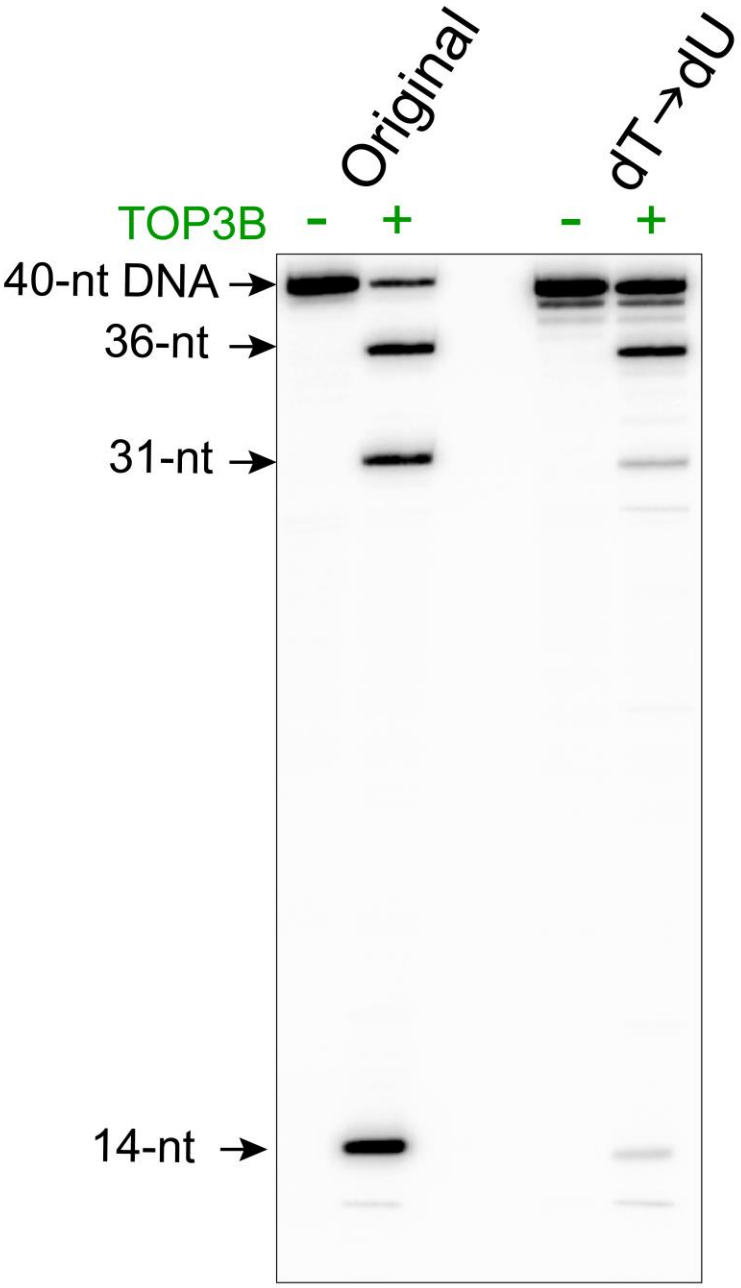

Supplementary figure 10. Thymine (T) to uracil (U) base substitutions within a 40-nt DNA substrate does not significantly alter the position of cleavage sites by TOP3B. DNA analog (dT-> dU) was generated by replacing all the thymine bases with uracil bases without changing the DNA backbone. TOP3B cleavage products with original DNA substrate are indicated. Assay was repeated twice independently with similar results.

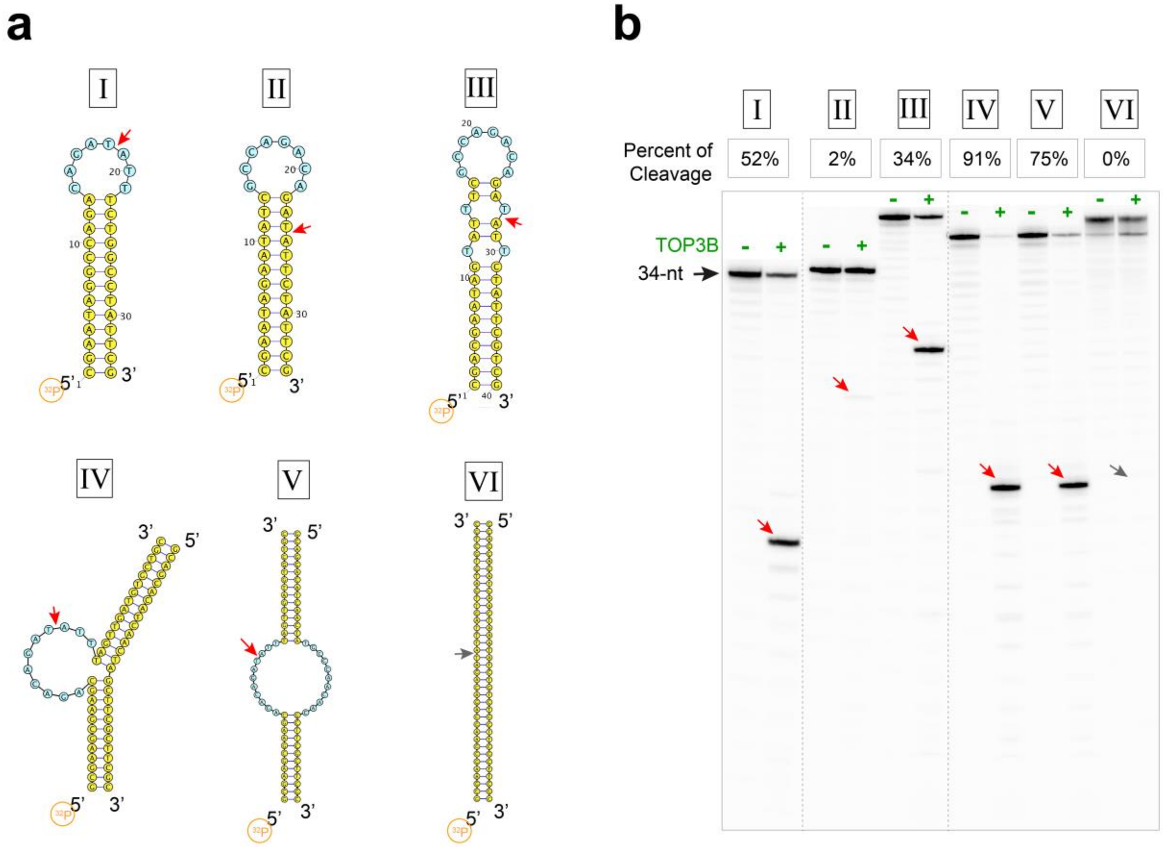

Supplementary figure 11. TOP3B requires single-stranded nucleic-acid substrates for cleavage. a, DNA oligonucleotides (I-VI) with different secondary-structure elements all contain a strong TOP3B recognition site: 5'-CAGACAGAT*ATT-3' where* indicates the TOP3B cleavage site. Substrates with predicted secondary structures were drawn with the RiboSketch software and red/grey arrows indicate the position of the TOP3B cleavage sites in the substrates. Orange circles represent ^{32}P labels at the indicated DNA ends. **b**, TOP3B cleavage assays with the indicated substrates (I-VI). Red arrows indicate TOP3B cleavage products generated by cleavage at the corresponding sites shown in **a**. The grey arrow indicates lack of cleavage at the indicated site in **a**. Overall percent of DNA cleavage (%) were obtained by quantification of the DNA substrate bands without and with TOP3B addition. Grey dotted line represents the combination of different lanes on the same gel with the same exposure time. Assay was repeated twice independently with similar results.

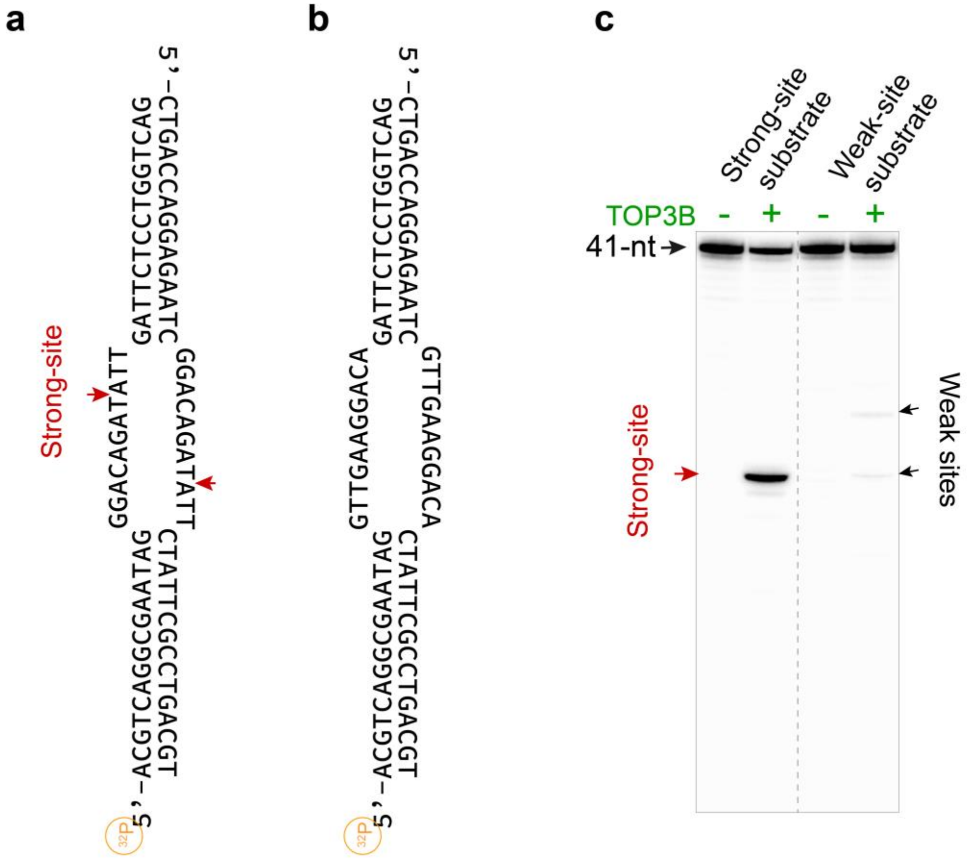

Supplementary figure 12. Design of the DNA mismatch bubble substrates containing a strong or weak TOP3B recognition site used for single-molecule analysis. a, DNA insert with an 11-base mismatch bubble containing a strong TOP3B site on each DNA strand (indicated by arrows). **b,** DNA insert with an 11-base mismatch bubble lacking a strong TOP3B site. **c,** Gel-based DNA cleavage assay using the ^{32}P-labeled DNA inserts as substrates confirmed the differential TOP3B cleavage profiles with these designed DNA inserts. Grey dashed line indicates a combination of different lanes on the same gel with the same exposure time. Cleavage assay was repeated twice independently with similar results.

Supplementary References

1. Saha, S. et al. DNA and RNA Cleavage Complexes and Repair Pathway for TOP3B RNA- and DNA-Protein Crosslinks. *Cell Rep* **33**, 108569 (2020).
