# Supplementary material for: Structural and biochemical basis for DNA and RNA catalysis by human Topoisomerase 3β
Source: Nat Commun. 2022 Aug 9;13:4656. doi: 10.1038/s41467-022-32221-3 (PMC9363430; doi:10.1038/s41467-022-32221-3)
Supplement: Supplementary file 3 — Reporting Summary [file 41467_2022_32221_MOESM3_ESM.pdf]

nature portfolio

Corresponding author(s): Yves Pommier

Last updated by author(s): May 12, 2020

Reporting Summary

Nature Portfolio wishes to improve the reproducibility of the work that we publish. This form provides structure for consistency and transparency in reporting. For further information on Nature Portfolio policies, see our Editorial Policies and the Editorial Policy Checklist.

Statistics

For all statistical analyses, confirm that the following items are present in the figure legend, table legend, main text, or Methods section.

n/a	Confirmed	
☐	☒	The exact sample size (*n*) for each experimental group/condition, given as a discrete number and unit of measurement
☐	☒	A statement on whether measurements were taken from distinct samples or whether the same sample was measured repeatedly
☒	☐	The statistical test(s) used AND whether they are one- or two-sided<br>*Only common tests should be described solely by name; describe more complex techniques in the Methods section.*
☒	☐	A description of all covariates tested
☐	☒	A description of any assumptions or corrections, such as tests of normality and adjustment for multiple comparisons
☐	☒	A full description of the statistical parameters including central tendency (e.g. means) or other basic estimates (e.g. regression coefficient) AND variation (e.g. standard deviation) or associated estimates of uncertainty (e.g. confidence intervals)
☒	☐	For null hypothesis testing, the test statistic (e.g. *F*, *t*, *r*) with confidence intervals, effect sizes, degrees of freedom and *P* value noted<br>*Give P values as exact values whenever suitable.*
☒	☐	For Bayesian analysis, information on the choice of priors and Markov chain Monte Carlo settings
☒	☐	For hierarchical and complex designs, identification of the appropriate level for tests and full reporting of outcomes
☒	☐	Estimates of effect sizes (e.g. Cohen's *d*, Pearson's *r*), indicating how they were calculated

Our web collection on statistics for biologists contains articles on many of the points above.

Software and code

Policy information about availability of computer code

Data collection	Acrylamide gels containing 32P-labeled DNA/RNA oligos were scanned with GE Typhoon Phosphorimager using Typhoon FLA 9500 Control Software. Single-molecule DNA extension tracking were carried out using the Xvin software suite (PicoTwist SARL).
Data analysis	For single-molecule data processing: changes of DNA extension and dwell times were measured with Xvin software suite (PicoTwist); Histogram plots of step–amplitude/step-dwell time distributions and Gaussian/exponantial-decay fitting were carried out with Xvin software suite (PicoTwist). Quantification of DNA/RNA bands on the acrylamide/agarose gels were carried out with ImageJ v1.46 software. Illustrations were generated with Illustrator CC (Adobe), UCSF Chimera v1.15, PyMOL v2.5, Ribosketch v0.8.2 and WebLogo online server (UC berkeley).

For manuscripts utilizing custom algorithms or software that are central to the research but not yet described in published literature, software must be made available to editors and reviewers. We strongly encourage code deposition in a community repository (e.g. GitHub). See the Nature Portfolio guidelines for submitting code & software for further information.

Data

Policy information about availability of data

All manuscripts must include a data availability statement. This statement should provide the following information, where applicable:

- Accession codes, unique identifiers, or web links for publicly available datasets
- A description of any restrictions on data availability
- For clinical datasets or third party data, please ensure that the statement adheres to our policy

The supportive data for this study are available from the corresponding authors upon reasonable request.

Field-specific reporting

Please select the one below that is the best fit for your research. If you are not sure, read the appropriate sections before making your selection.

☒ Life sciences ☐ Behavioural & social sciences ☐ Ecological, evolutionary & environmental sciences

For a reference copy of the document with all sections, see nature.com/documents/nr-reporting-summary-flat.pdf

Life sciences study design

All studies must disclose on these points even when the disclosure is negative.

Sample size	We chose the sample sizes in our single-molecule measurements that are sufficient for constructing histograms and Gaussian/exponential decay fitting, in order to obtain a mean value for each dataset and to show significance between indicated datasets in the manuscript.
Data exclusions	No data were excluded from the analyses.
Replication	All the gel based assays and single-molecule experiments were conducted independently for at least twice with similar results. Numbers of replication were mentioned in the manuscript.
Randomization	Randomization is not relevant this study as this study did not involve sample/organisms/participants allocation.
Blinding	Blinding is not relevant this study as this study did not involve sample/organisms/participants allocation.

Reporting for specific materials, systems and methods

We require information from authors about some types of materials, experimental systems and methods used in many studies. Here, indicate whether each material, system or method listed is relevant to your study. If you are not sure if a list item applies to your research, read the appropriate section before selecting a response.

Materials & experimental systems

n/a	Involved in the study	
☐	☒	Antibodies
☐	☒	Eukaryotic cell lines
☒	☐	Palaeontology and archaeology
☒	☐	Animals and other organisms
☒	☐	Human research participants
☒	☐	Clinical data
☒	☐	Dual use research of concern

Methods

n/a	Involved in the study	
☒	☐	ChIP-seq
☒	☐	Flow cytometry
☒	☐	MRI-based neuroimaging

Antibodies

Antibodies used	Anti-TOP3B antibody [EP7779] - C-terminal, abcam (ab183520).
Validation	Anti-TOP3B antibody (ab183520) was validated by the manufacturer for detecting Mouse, Rat and Human topoisomerase 3B enzymes.

Eukaryotic cell lines

Policy information about cell lines

Cell line source(s)	HEK293T cell line was purchased from Thermo Fisher Scientific.
Authentication	Not authenticated.
Mycoplasma contamination	Not tested.
Commonly misidentified lines (See ICLAC register)	No misidentified lines used.
